# Protocol for a systematic review of methods and cost-effectiveness findings of economic evaluations of obesity prevention and/or treatment interventions in children and adolescents

**DOI:** 10.1186/s13643-018-0718-5

**Published:** 2018-04-02

**Authors:** Mandana Zanganeh, Peymane Adab, Bai Li, Emma Frew

**Affiliations:** 1 Institute of Applied Health Research, College of Medical and Dental Sciences, University of Birmingham, Birmingham, UK; 2 Health Economics Unit, Institute of Applied Health Research, College of Medical and Dental Sciences, University of Birmingham, Birmingham, UK

**Keywords:** Economic evaluation, Obesity, Children, Adolescents, Prevention, Treatment, Intervention

## Abstract

**Background:**

Childhood obesity is a major global public health problem, with governments increasingly having to undertake various strategies to reduce excess weight in their populations. Considering the increasing number of well-conducted intervention studies in the field of childhood obesity prevention, there are relatively few published economic evaluations. The proposed systematic review will explore the methods of these economic evaluations, examine the limitations and establish the evidence base for cost-effectiveness analyses.

**Methods/design:**

Systematic review methodology will be applied to identify, select and extract data from published economic evaluation studies (trial-based, non-trial based, simulation-based, decision model and trial based model economic evaluations) of obesity prevention and/or treatment interventions in children and adolescents. A systematic literature search will be conducted using bibliographic databases (MEDLINE, EMBASE, CINAHL, Web of Science, EconLit, PsycINFO, Cochrane Library, Centre for Reviews and Dissemination (CRD) and Cost-Effectiveness Analysis (CEA) Registry). The review will only include full economic evaluations. There will be no restrictions based on language, perspective, follow-up duration, sample size, country or setting. To minimise selection bias, translation of non-English language articles will be undertaken. The quality of included studies will be assessed. Following data extraction, a narrative synthesis of the results from the included studies will be undertaken. Subgroup analysis will be considered where deemed appropriate.

**Discussion:**

The findings from this review, which will include primary studies, will provide evidence to assist health policy decision makers interpret economic evaluations in this field. In addition, we will identify gaps in the current literature to inform future-related research.

**Systematic review registration:**

Prospero CRD42017062236

**Electronic supplementary material:**

The online version of this article (10.1186/s13643-018-0718-5) contains supplementary material, which is available to authorized users.

## Background

Childhood obesity is a major global public health problem, which leads to health, social and emotional problems, as well as associated high health care costs [1]. Thus, governments and policy makers are increasingly seeking to address this issue through a variety of strategies [2]. Over the last 3 decades, the percentage of people who are overweight or obese has been increasing globally [3]. Estimated age-standardised prevalence of obesity in children and adolescents in 2016 ranged from higher than 30% in, for example, Nauru, the Cook Islands, and Palau to lower than 2% in other countries, including Ethiopia [4]. Obesity is as much an issue in developing as in developed countries. Although the prevalence of childhood obesity may be higher in developed countries, the rate of increase over the last decade is steeper in many developing countries [4].

Children who are overweight or obese run an increased risk of becoming obese in adulthood with the associated adverse health effects. However, even during childhood, they are more likely to develop early symptoms and signs of co-morbidities, hypertension and insulin resistance [5]. The increasing prevalence of childhood obesity is also a major economic concern. Obesity leads to significant economic and societal consequences via both direct and indirect costs [6, 7]. Directs costs relate to the healthcare needs arising from the related health problems, whilst indirect costs, which are estimated to even exceed the direct costs, are a result of productivity losses (sick leave, disability, premature death) [7, 8]. Also, lower academic achievement among children with overweight and obesity could hinder their future employment prospects [5]. Obesity prevention and treatment in children and adolescents is therefore a key public health priority. However, despite the increasing number of intervention studies, there are relatively few published economic evaluations [9–11].

The concept of opportunity cost plays a fundamental role in the economist’s view of costs. In many countries, the scarcity of economic resources relative to needs requires decision makers to prioritise spending in the knowledge that the use of resources in one way prevents their use in others [12]. They need to invest in initiatives which offer the best value for money from increasingly limited public resources [13]. Economic evaluation is a means to aid decisions about public resource allocation to maximise society’s welfare [3, 13]. To reflect a societal cost, where possible, all resource use should be measured, outlining how costs fit within a given sector, such as health, transport, education or the wider community [14]. The UK Treasury guidance distinguishes between reporting accounting costs, to understand the flow of expenditure and resources, and reporting opportunity costs, reflecting the wider costs and benefits of interventions [15]. Opportunity cost can be assessed directly with cost utility or cost-effectiveness studies [12].

Although the concept of opportunity cost is important, there can be a number of challenges with applying it within the context of economic evaluation [12]. For example, the perspective of the study is important as this determines which costs and effects are included and the choice of comparisons are crucial in that these reflect the outcomes forgone from alternative uses of the limited resources. However, when incorporating costs and outcomes that span across multiple sectors as is commonly the case within obesity interventions, it is not always clear what society’s willingness to pay is for a non-health effect caused by a health-focused intervention or funded from a ‘health care budget’, or what the opportunity cost is of non-health resources, e.g. the value of a pound within the education sector. Also, valuation of resources for which no market exists, such as informal care, or patient time costs, requires specific methods [12]. Due to such challenges, few studies are ever completely explicit about their estimates of opportunity costs.

Discounting enables a comparison between interventions that lead to benefits and incur costs over a number of years [16]. Currently, NICE recommends a discount rate of 3.5% per year for both costs and outcomes [17]. For childhood obesity prevention interventions, the effect of discounting will cause future health gains to be devalued [13]. However, failure to discount future benefits would consider interventions to be more cost effective than they would otherwise appear [18].

Seven recent reviews [5, 19–24] have summarised the cost-effectiveness of obesity prevention and/or treatment interventions in young people; however, none have used or reported rigorous methods for conducting their reviews. Only two of them consider accounting for costs occurring across different sectors [5, 23]. Five of the previous reviews had restrictions based on language criteria [5, 19–21, 23] and four of the previous reviews explicitly excluded studies that were conducted in developing countries within their exclusion criteria [5, 19, 21, 23]. Only three of the reviews used established criteria, e.g. Drummond or Philips to assess the quality of the primary studies, and used preventative steps to minimise bias and errors in the quality assessment process [5, 20, 23]. The search strategy was adequate and appropriate in over half of the reviews (4/7) [5, 20, 22, 23]. However, several more relevant databases could have been searched to ensure that all studies were identified. Further, the latest date that the previous published reviews covered was up to November 2015 and, since then, at least 3 new economic evaluation studies of childhood obesity interventions have been published [25–27].

One recent review paper on child and adolescent obesity summarised the findings from 21 obesity prevention studies that included economic evaluation [24]. The authors found that 6 interventions were dominant, in that the interventions are likely to result in both health gains and real cost savings to health services or to society; 12 were likely to be cost-effective, in that financial costs are worthwhile for the health gains and 3 were unlikely to be cost-effective. However, this brief summary did not critique the methods used for economic evaluation.

A further review of 8 cost-effectiveness analysis studies of childhood obesity prevention, which included a subset of the 21 studies reviewed in the other review, found that 1 intervention was dominant; 4 were likely to be cost-effective and 3 were unlikely to be cost-effective [20]. However, this review also did not provide information regarding how opportunity cost was valued, and what perspective was adopted for economic evaluation.

A review of the methods used within 6 published economic evaluations of obesity prevention studies in early childhood (preschool children (< 6 years)) limited inclusion criteria to trials that included specific outcome measures (at least one of the following: BMI, waist circumference or overweight prevalence) [5]. Furthermore, all included studies were based on behavioural interventions that targeted both diet and physical activity but none that targeted either diet or physical activity alone. It found that 3 interventions were cost-effective and 1 was not cost-effective.

Another review focused on economic evaluations of physical activity programmes as a primary prevention approach for obesity in children and adolescents [23]. This did not provide detailed information about the numbers of interventions that were cost-effective. A review of childhood obesity interventions in Australia summarised the findings from 13 community, behavioural, pharmaceutical and surgical interventions and assessed their cost-effectiveness [19]. In line with the Assessing Cost-Effectiveness (ACE) obesity approach [28], all included studies followed the same methodology for the economic evaluation allowing a comparison of cost-effectiveness between interventions. Similar to the previous reviews, the authors did not critically assess the methods used for economic evaluation.

A similar approach to the Australian review was applied in another review, in which the ACE approach was adapted to the US setting [21]. Purposive sampling of intervention studies was applied to represent a ‘broad range of national scalable interventions’, including 4 intervention studies. Another review focused on cost of illness and cost-effectiveness of childhood obesity interventions [22]. This review also did not provide information regarding the methods to value opportunity cost, or the perspective for the evaluation.

In summary, none of the existing reviews considered issues associated with the characteristics of the economic methods used, such as methods for collecting resource use data, how opportunity cost has been captured, reporting the largest cost drivers which impact upon cost-effectiveness and critiquing the modelling methods where appropriate. Also, there are no review studies addressing the question of how methods vary by setting, country, type of intervention or over time. These issues are important to consider when reviewing cost-effectiveness evidence [16]. It is for these reasons that a new systematic review will be conducted. The aim of this review is to critique the methods used in economic evaluations of child and adolescent obesity and summarise cost-effectiveness evaluations of prevention and treatment interventions. Primary economic evaluation studies will be included, with no restrictions on the setting, language or country.

## Methods/design

### Objectives (s)

The primary objective is to systematically review methods of economic evaluations of obesity prevention and/or treatment interventions in children and adolescents. The review will examine the limitations and establish the evidence base for cost-effectiveness, focusing on methods for collecting resource use, valuing opportunity costs, methods for reporting costs and effectiveness across health and non-health settings, measures of effectiveness, and where appropriate, a critical review of decision-analytic models.

A secondary objective is to determine how the methods (in terms of perspective, costs included, outcomes and method of assessing cost-effectiveness) vary by setting, country, type of intervention or time period of the study with reference to any guidelines from the country in which the study is set.

The guidelines by the Cochrane Collaboration for Reviews and Centre for Reviews and Dissemination (CRD) will be followed in the systematic review [29, 30]. The systematic review protocol has been registered with the international prospective register of systematic reviews (PROSPERO) database ref. (CRD42017062236) and has been reported according to the PRISMA-P guidelines (see Additional file 1).

### Selection criteria

Since the objective of the systematic review will be to critique the methods used in economic evaluations of child and adolescent obesity and summarise cost-effectiveness evaluations of prevention and treatment interventions, studies will be included in the review if they meet the following criteria:

#### Types of study to be included

Full economic evaluations (studies in which both the costs and outcomes of the alternatives are examined and in which a comparison of two or more interventions or case alternatives is undertaken) including:

- ◦ Primary economic evaluation studies including:
  - Trial-based
  - Non-trial based
  - Simulation-based
  - Decision model
  - Trial-based model

Qualitative studies, conference abstracts, short notes, comments, editorials and study protocols will be excluded. Partial economic evaluations will also be excluded because the review will synthesise the evidence base for cost-effectiveness and provide an assessment of methods for full economic evaluations.

#### Condition or domain being studied

Condition or domain being studied is overweight or obesity in children and adolescents.

#### Participants/population

Children and adolescents aged 0–19 years at the start of the intervention and/or their parents/guardians will be included. Family based interventions will be included, in which case, the target participants will be the children. There will be no restrictions on participant characteristics such as gender, baseline weight status or country. Both developed and developing countries will be included. Any interventions to tackle obesity due to a secondary cause (i.e. Prader-Willi syndrome) will not be included.

#### Intervention(s) and exposure(s)

Any behavioural, community, policy or environmental interventions aimed at the treatment or prevention of overweight/obesity in children and/or adolescents will be included. Considering that prevention and treatment interventions are likely to be diverse the findings will be synthesised separately for both types of interventions and compared and contrasted if relevant. Pharmacological or surgical interventions will not be included.

#### Comparator(s)/control

There will be no restrictions on the types of comparator(s). For example, the comparator can be either no intervention or another intervention. However, the study should have a clear definition of the comparison.

#### Outcome(s)

##### Primary outcomes

There will be no restrictions on study outcomes because the purpose of the review is to assess what outcomes are reported within economic evaluations. However, potentially relevant primary outcomes will be disability-adjusted life years (DALYs), quality-adjusted life years (QALYs), effectiveness outcomes such as kilogramme (kg) weight loss, % body fat, BMI (*z* score), waist circumference, skinfold thickness, overweight and obesity case avoided, additional minute of MVPA, increase in physical activity and MET hour gained.

##### Secondary outcomes

All outcomes as mentioned above.

### Other criteria

There will be no restrictions based on language, perspective, follow-up duration, sample size or setting. Studies will be sought if published between January 2001 and the date of the searches. The year 2001 is chosen since the first study evaluating the cost-effectiveness of a childhood obesity treatment intervention was published in 2001, followed 2 years later by the first economic evaluation of a childhood obesity prevention intervention [22]. The searches will be re-run just before the final analyses and further studies retrieved for inclusion.

### Search strategy

The following electronic bibliographic databases of published studies will be searched:

- MEDLINE (Ovid)
- EMBASE (Ovid)
- Web of Science
- CINAHL Plus
- EconLit
- PsycINFO
- Cochrane Central Register of Controlled Trials (CENTRAL) and Cochrane Database of Systematic Reviews (CDSR)
- Centre for Reviews and Dissemination (CRD) Databases (Database of Abstracts of Reviews of Effects (DARE), the National Health Service Economic Evaluation Databases (NHS EED), Health Technology Assessment Database (HTA))
- Cost-Effectiveness Analysis (CEA) Registry

In addition, the following sources will also be used to identify potential additional studies, including grey literature sources for review:

- Citation tracking in Google Scholar to retrieve further references.
- Searching the relevant National Institute for Health and Care Excellence (NICE) guidelines.
- The systematic scanning of the reference lists of eligible studies and review articles.
- Contact with study authors where appropriate.
- Grey literature such as OpenSIGLE, National Obesity Observatory, NHS Evidence, National Technical Information Service, Healthcare Management Information Consortium (HMIC) and RePEC (Economic Working papers) database.

### Potential search terms

The potential search terms are systematic review, economic evaluation, cost-effectiveness, obesity, children, adolescents, prevention, treatment and intervention.

A Subject Advisor for Medicine and an Information Specialist applied a search strategy for MEDLINE, which was based on medical subject headings (MeSH) terms and text words of key papers that were identified beforehand (see Additional file 2). The search terms and text words will be adapted for use with other bibliographic databases.

The literature search results will be managed using Endnote 7 (Thomson Reuters) to facilitate removal of duplicate records, study selection, recording decisions and references.

### Study selection procedure

The review will follow a two-stage method. Paper selection will be based on the inclusion and exclusion criteria:

- First, two reviewers will independently screen titles and abstracts against the selection criteria.
- Second, where there is doubt, the full-text version will be requested. All full-text papers will be further reviewed by two reviewers and a final decision made with respect to the inclusion/exclusion criteria.

Any disagreement or conflicting views between the reviewers over the eligibility of specific studies will be resolved by discussion or the final judgement of a third reviewer. Both stages of the selection process will be piloted and if necessary modified.

A PRISMA flow diagram will be used to illustrate the study selection processes [31]. Details of articles excluded at the second stage will be recorded along with the reason for exclusion [31]. To aid study selection and analysis of non-English language articles, translation either in part or in whole will be undertaken by appropriate University members. Endnote will be used to keep track of references.

### Data extraction

Publication information, study characteristics and findings from the included studies, related to the research question, will be recorded in a standardised, pre-piloted data extraction form using Excel. Extracted information will include:

- Authors
- Publication year
- Country
- Currency unit
- Study design
- Setting
- Target population
- Sample size
- Overview and aim of the intervention
- Comparator
- Measures of effectiveness
- Model specification
- Study perspective
- Length of follow-up
- Time horizon
- Methods for collecting resource use
- Price year
- Costs categories
- Largest cost drivers
- Opportunity cost
- Excluded costs
- Discount rate
- Total/average intervention costs
- ICER
- Uncertainty analysis
- Sensitivity analysis
- Funding source

The main reviewer will extract the data. To validate the data extraction process, the process will be independently checked for completeness and accuracy by a second reviewer. Any discrepancies between the reviewers over the data extraction process will be identified and resolved by discussion or the final judgement of a third reviewer. Missing data will be requested from study authors.

### Quality assessment of included studies

For this review, the Drummond checklist is not comprehensive enough to assess the quality of the economic evaluation studies included [16]. However, the Philips checklist is only applicable for modelling studies [32]. Therefore, the quality assessment checklist developed by the Centre for Reviews and Dissemination of the University of York, which is a slightly adapted version of the Drummond checklist, will be used [33]. A few extra questions that are relevant to a paediatric setting will be added from the Paediatric Quality Appraisal Questionnaire (PQAQ) because this checklist is specially constructed for assessing the quality of measures for such a population [34]. The quality assessment will provide a systematic and critical descriptive overview of key methodological elements. The scoring of the papers will be divided into 4 categories: poor quality (scoring 40–55%), good quality (scoring 55–70%), very good quality (scoring 70–85%) and excellent quality (scoring 85% or higher). To validate the quality assessment process, the process will be independently checked for completeness and accuracy by a second reviewer.

### Strategy for data synthesis

Following data extraction, the reviewer will provide a narrative synthesis of the results from the included studies, structured around general characteristics, characteristics of the intervention programmes and economic findings (basic characteristics, study perspectives, resource use, cost categories, cost-effectiveness findings and sensitivity analysis), along with a critique of methods used for economic evaluation.

### Analysis of subgroups or subsets

Subgroup analysis will be considered where deemed appropriate.

### Reporting

The review and its findings will be reported in accordance to the PRISMA guidelines [31]. The strengths and weaknesses of the methods used for economic evaluation will be discussed. The implications of the review findings will be discussed within the context of current and future policy related to obesity.

## Discussion and potential impact

Seven recent reviews have summarised the cost-effectiveness of obesity prevention and/or treatment interventions in young people; however, none have used or reported rigorous methods for conducting their reviews. This comprehensive systematic literature review will provide a critical review of the methods used in economic evaluations of child and adolescent obesity and a narrative summary of reported cost-effectiveness evaluations of prevention and treatment interventions. This critical analysis of the economic evidence can inform policies for tackling childhood obesity. We anticipate that the review will also highlight gaps in the current literature to inform future economic evaluation research in this area.

## Additional files

Additional file 1: PRISMA-P 2015 checklist: recommended items to include in a systematic review protocol. (DOCX 23 kb)

Additional file 2: Sample search strategy from MEDLINE. (DOCX 13 kb)

## Abbreviation

BMI: Body mass index
CDSR: Cochrane Database of Systematic Reviews
CEAR: Cost-Effectiveness Analysis Registry
CRD: Centre for Reviews and Dissemination
DALYs: Disability-adjusted life years
DARE: Database of Abstracts of Reviews of Effects
HMIC: Healthcare Management Information Consortium
HTA: Health Technology Assessment Database
MeSH: Medical subject headings
MET: Metabolic equivalent
MVPA: Moderate to Vigorous Physical Activity
NHS EED: National Health Service Economic Evaluation Database
NHS: National Health Service
NICE: National Institute for Health and Clinical Excellence
PQAQ: Paediatric Quality Appraisal Questionnaire
QALYs: Quality-adjusted life years
