# Supplementary material for: Protocol for a systematic review of methods and cost-effectiveness findings of economic evaluations of obesity prevention and/or treatment interventions in children and adolescents
Source: Syst Rev. 2018 Apr 2;7:54. doi: 10.1186/s13643-018-0718-5 (PMC5879570; doi:10.1186/s13643-018-0718-5)
Supplement: Supplementary file 2 — Sample search strategy from MEDLINE. (DOCX 13 kb) [file 13643_2018_718_MOESM2_ESM.docx]

**Additional file 2: Sample search strategy from MEDLINE**

**MEDLINE (Ovid)**

1. exp Obesity/
2. Obese.mp.
3. exp Overweight/
4. (BMI or body mass index).af.
5. Weight gain/
6. (Overweight or over weight or obesity or adipose).af.
7. exp Child/
8. exp Infant/
9. (Child* or adolescen* or infant*).af.
10. Schoolchild*.mp.
11. exp Adolescent/
12. (Boys or girls or youth or youths).af.
13. (Teenage* or young person).af.
14. (Nutrition adj2 intervent*).af.
15. (Obesity adj2 prevent* or treat*).af.
16. Counsel?ing.mp.
17. exp support groups/
18. exp Health Behaviour.mp.
19. exp Life Style/
20. exp Delivery of Health Care/
21. exp Social Support/
22. exp Family Practice/
23. exp Parent-Child Relations/
24. Food Habits .mp.
25. exp Diet therapy/
26. exp Food Preferences/
27. exp Exercise therapy/
28. Physical activit*.mp.
29. Economic Evaluat*.mp.
30. Cost* .ti.
31. Cost?Benefit*.mp.
32. Cost?Utilit*.mp.
33. Cost?Effective*.mp.
34. exp "costs and cost analysis"/
35. 1 or 2 or 3 or 4 or 5 or 6
36. 7 or 8 or 9 or 10 or 11 or 12 or 13
37. 14 or 15 or 16 or 17 or 18 or 19 or 20 or 21 or 22 or 23 or 24 or 25 or 26 or 27 or 28
38. 29 or 30 or 31 or 32 or 33 or 34
39. 35 and 36 and 37 and 38
40. Limit 39 to (yr=”2001-Current”)
